# A proposed nine-domain nutrient-function architecture for modelling adult nutrient adequacy across dietary preference variants and calorie tiers

**DOI:** 10.3389/fnut.2026.1923749

**Published:** 2026-08-25

**Authors:** Vivek Singh

**Affiliations:** Healthisnow Consultancy Pvt Ltd, New Delhi, India

**Keywords:** dietary modelling, dietary pattern, dietary preferences, food fortification, maintenance diet, micronutrient adequacy, nutrient adequacy, nutrient-function domains

## Abstract

**Background:**

Dietary labels, calorie targets and broad food-group advice do not by themselves show whether nutrient functions are preserved when foods are excluded or substituted. This study developed a proposed nine-domain nutrient-function architecture to translate adult nutrient requirements into auditable food-source decisions across contrasting dietary preferences and calorie tiers.

**Methods:**

The architecture was developed through a design-based, criterion-referenced process beginning with the declared nutrient, dependency, safety and implementation requirement universe; mapping recurrent substitution failures; clustering failures by shared planning action; and applying eight domain-retention criteria. Eleven source-availability archetypes were crossed with four maintenance calorie-tier anchors to generate 44 internal model-evaluation scenarios. Foods consumed periodically were converted to average daily equivalents. Candidate food quantities were iteratively reconciled against tier energy bands, benchmark adequacy, domain coverage, source dependencies, upper-limit screens and practical portions. Compatible ordinary foods were considered first; unresolved gaps or narrow-source vulnerabilities then triggered evaluation of alternative foods, verified fortification, targeted sources or conditional interpretation.

**Results:**

The model generated 1,012 non-energy scenario-nutrient rows. Sodium was retained as a safety/contextual output, leaving 22 ordinary adequacy nutrients and 968 ordinary adequacy rows. Final evaluated scenarios met a mean of 20.64 of 22 ordinary nutrients, with a scenario range of 19–21 and variant means of 20.25–21.00. Tier means increased from 20.00 in Lite to 21.00 in High-Output Maintenance. Iron and zinc each produced eight near-adequacy findings; no ordinary nutrient produced a moderate or major gap. Vitamin D remained conditional in all scenarios. Fortified plant milk, fortified nutritional yeast, algal EPA + DHA, iodized salt and a capped selenium source materially affected selected outputs. The safety screen produced 484 no-UL, 374 within-UL, 47 watch and 151 exceedance flags; most exceedance flags involved form-sensitive vitamin A, niacin and folate comparisons or sodium. Thirty-three scenarios showed moderate and 11 high sensitivity to the tested assumptions.

**Conclusion:**

The proposed architecture generated broad modelled benchmark coverage while making compensatory sources, conditional findings, source-form safety issues and broader food-matrix pathways visible. These internally constructed scenarios demonstrate feasibility under declared assumptions, not independent validation, clinical efficacy, real-world adherence or universal individual sufficiency.

## 1 Introduction

Adequate energy intake does not necessarily imply adequate nutrient intake. Diets can provide enough calories while remaining inadequate in vitamins, minerals, essential fatty acids, fibre or other food-derived constituents relevant to physiological function. Global modelling has identified widespread inadequate intakes of multiple micronutrients, while comparative dietary-risk analyses and cohort evidence associate poor diet quality with substantial non-communicable disease burden (1–3). These observations support direct examination of nutrient adequacy rather than inference from energy intake, macronutrient balance or the name of a dietary pattern.

Dietary preference adds a second layer of complexity. Omnivorous, fish-free, dairy-free, egg-free, vegetarian and vegan patterns can all be nutritionally adequate when appropriately planned (4–6). However, exclusion of a food category can remove several nutrient functions simultaneously. Removing fish changes preformed EPA + DHA, vitamin D, vitamin B12, iodine and selenium pathways; removing dairy changes calcium, riboflavin, vitamin B12 and fortified vitamin D pathways; removing all animal-source foods increases dependence on verified vitamin B12 sources and may heighten iron and zinc bioavailability concerns. The relevant scientific question is therefore not whether one dietary identity is inherently superior, but whether the functions carried by excluded foods are identified, restored where feasible and disclosed when restoration remains conditional.

This problem is not solved by calories or macronutrients alone. Macronutrient-focused weight-loss patterns can differ materially in micronutrient quality, and example menus from popular diet plans have not consistently provided complete micronutrient coverage at typical energy intakes (7, 8). Nor is the problem solved by replacing one food with another that appears similar at the culinary level. Replacing fish only with another protein may preserve protein but not preformed EPA + DHA; replacing milk with an unfortified plant beverage may preserve meal format but not calcium or vitamin B12; replacing iodized salt with a specialty non-iodized salt may preserve taste but not iodine. A substitution can therefore be acceptable by preference while being incomplete by nutrient function.

Food-based dietary guidelines translate nutrient requirements into locally meaningful foods, food groups and dietary patterns, while diet-quality indices such as the Healthy Eating Index and Diet Quality Index-International evaluate alignment, variety, adequacy, moderation and balance (9–11). Dietary records, recalls and technology-enabled intake tools estimate what people consume. These approaches are essential, but they primarily guide populations, score observed patterns or quantify intake. They do not fully specify a construction rule for preserving source-specific nutrient functions when a target dietary pattern must be rebuilt across different food exclusions, calorie requirements and cultural preferences.

The proposed nine-domain nutrient-function architecture addresses this upstream construction problem. It does not replace conventional nutrient classifications, claim to be an official dietary guideline or assume that nine is the uniquely correct number of domains. Instead, it translates nutrient requirements and recurrent substitution failures into nine recurring planning checkpoints. The checkpoints cover protein quality; essential fats and preformed long-chain omega-3 fatty acids; fibre-rich plant energy; leafy-green and phytonutrient exposure; nut, seed and trace-mineral sources; water-soluble and one-carbon nutrients; calcium, vitamin D and vitamin B12 source dependency; iodine and electrolyte control; and hydration and practical dietary implementation. Foods receive a primary planning role, while their full nutrient composition is counted across the complete food-by-nutrient matrix.

The architecture is intended for a recurring dietary cycle rather than a requirement that every nutrient source appear in every meal. Fish, eggs, legumes, dairy, meat, vegetables, nuts, seeds and fermented foods can rotate across the week, provided their periodic quantities are converted consistently and the nutrient functions of the complete pattern remain visible. This creates the possibility that a compact one- or two-meal daily structure can still carry broad nutrient-function coverage when foods are deliberately combined across the recurring week. Meal frequency itself, however, is not tested in this study.

The model also distinguishes two levels of nutritional breadth. The first is a quantified benchmark and safety layer covering energy, protein, fibre, vitamins, minerals, ALA and EPA + DHA. The second is a broader food-matrix layer represented by whole grains, legumes, roots, vegetables, leafy greens, fruits, nuts, seeds and fermented foods. These foods may provide carotenoids, phenolic acids, flavonoids, lignans, phytosterols, resistant starch, oligosaccharides and fermentation-derived components. Because many such compounds lack agreed intake benchmarks and vary substantially by food species and processing, they are treated as structurally preserved exposure pathways rather than formal adequacy endpoints.

The numerical analysis is anchored to Indian adult reference values, Indian dietary-guidance context and Indian food-composition data, with selected international contextual targets or upper-limit references used where required (12–15). Eleven source-availability archetypes were combined with four maintenance calorie-tier anchors to generate 44 internal model-evaluation scenarios. The study asked whether the proposed architecture could be operationalized consistently across these source restrictions and energy levels while making adequacy, post-gap restoration, conditionality, safety and assumption sensitivity explicit. It did not test clinical outcomes, disease reversal, adherence, biomarkers or therapeutic efficacy.

## 2 Materials and methods

### 2.1 Study design, reference frame and scope

This was a deterministic dietary modelling study of a proposed nutrient-function architecture. It used constructed adult maintenance patterns rather than participant intake records, clinical interventions or biomarker observations. The purpose was to determine whether a constant set of food-source planning functions could be operationalized across contrasting dietary source restrictions and calorie-tier anchors under transparent quantitative assumptions.

The numerical layer used Indian-reference nutrient benchmarks and food-composition data. ICMR-NIN nutrient requirements and Indian dietary guidance provided the primary adult reference context, and Indian Food Composition Tables provided the principal composition layer (12–14). Where the adult male and female reference values differed, the higher adult value was used as a conservative planning comparator. Selected international references were used for nutrients or safety limits not fully represented in the principal Indian benchmark set, including potassium, vitamin E, vitamin K, ALA, EPA + DHA and selected upper-limit interpretations (15–24). The study therefore tests an Indian-reference implementation of the architecture, not a jurisdiction-free universal benchmark model.

The target population frame was stable, non-pregnant, non-lactating adults in a maintenance context. Children, adolescents, pregnancy, lactation and clinical conditions requiring individualized medical nutrition therapy were outside scope. The model did not prescribe medication changes, diagnose deficiency or estimate clinical risk.

### 2.2 Architecture-development protocol

The nine-domain architecture was developed through a design-based, criterion-referenced conceptual synthesis. It was not derived from a national dietary survey, statistical factor analysis, Delphi consensus panel or pre-existing diet-quality index. Its purpose was to translate conventional nutrient requirements and known source vulnerabilities into food-source planning decisions that remain visible when foods are excluded, substituted or scaled.

Development proceeded through nine linked stages. First, the intended use was defined as preference-sensitive adult maintenance pattern construction and audit. Second, the requirement universe was declared: energy-tier compatibility; protein and fibre; benchmarked vitamins and minerals; essential fatty acids; narrow-source nutrients; bioavailability cautions; upper-limit and sodium constraints; hydration and implementation needs; and broader food-matrix exposures. Third, recurrent exclusion-related failure modes were mapped. These included loss of EPA + DHA when fish was replaced only as protein; loss of calcium, vitamin B12 or fortified vitamin D when dairy was removed; loss of iodine when iodized salt was replaced; reduction of iron and zinc bioavailability in plant-heavy patterns; displacement of vegetables by energy- or protein-dense foods; and excessive exposure when concentrated selenium or other nutrient sources were duplicated.

Fourth, failure modes were clustered where they required the same primary planning action and kept separate where source pathways, scaling behaviour, fortification dependency, bioavailability or safety constraints differed materially. Fifth, candidate domains were retained only when they passed the predefined retention criteria. Sixth, foods were assigned a primary planning role without restricting their total nutrient contribution. Seventh, periodic foods were converted to recurring-week average daily equivalents. Eighth, the same construction rules were applied across 44 source-restriction and calorie-tier scenarios. Ninth, the validation boundary was declared: the exercise was an internal feasibility and stress test, not external or construct validation.

### 2.3 Domain-retention and delimitation criteria

Eight criteria governed domain retention. A domain had to: (1) protect at least one declared nutrient, safety or implementation function; (2) represent a distinct planning decision that could not be merged without hiding a materially different source or safety issue; (3) be vulnerable to preference-based substitution; (4) produce a quantity, adequacy output or explicit flag that could be audited; (5) operate across daily or periodic foods within a recurring week; (6) permit culturally variable food sources; (7) expose rather than conceal unresolved dependencies; and (8) remain extensible to broader food-component mapping without converting non-benchmarked compounds into false RDA/EAR outcomes.

Domain boundaries were functional rather than biochemical. Foods can contribute to several domains, but each input was assigned one primary planning role to prevent the same food from being treated as multiple independent requirements. All nutrients in that food were nevertheless credited globally in the nutrient calculation. Thus, fish assigned to the fatty-acid domain still contributed protein, vitamin B12, vitamin D, iodine and selenium; dairy assigned to the calcium-B12-vitamin D domain still contributed protein and riboflavin; and legumes assigned to the plant-energy/fibre domain still contributed protein, iron, zinc, folate and magnesium.

The nine-domain partition was retained as a parsimonious operational prototype. The study does not claim that nine is mathematically optimal. Future construct-validation work may merge, divide or reassign domains and compare alternative structures.

### 2.4 Operational nine-domain structure

The domains can be understood in three functional layers. The first layer protects foundational substrate and energy functions. Domain 1 preserves sufficient preference-compatible protein and essential amino-acid logic. Domain 2 separates general fat provision from ALA and preformed EPA + DHA decisions so that fish removal is not treated merely as protein substitution. Domain 3 supplies scalable plant energy, dietary fibre, potassium, magnesium and food volume through grains, pseudocereals, legumes, roots, tubers and related foods.

The second layer protects micronutrient density and broader food-matrix exposure. Domain 4 preserves leafy-green and vegetable density so that these foods are not displaced by protein- or energy-dense choices. Domain 5 provides controlled nut, seed, vitamin E and trace-mineral pathways while preventing concentrated selenium sources from being treated as unlimited. Domain 6 makes thiamine, riboflavin, niacin, vitamin B6, folate, choline-related and one-carbon nutrient pathways explicit.

**Figure 1:**
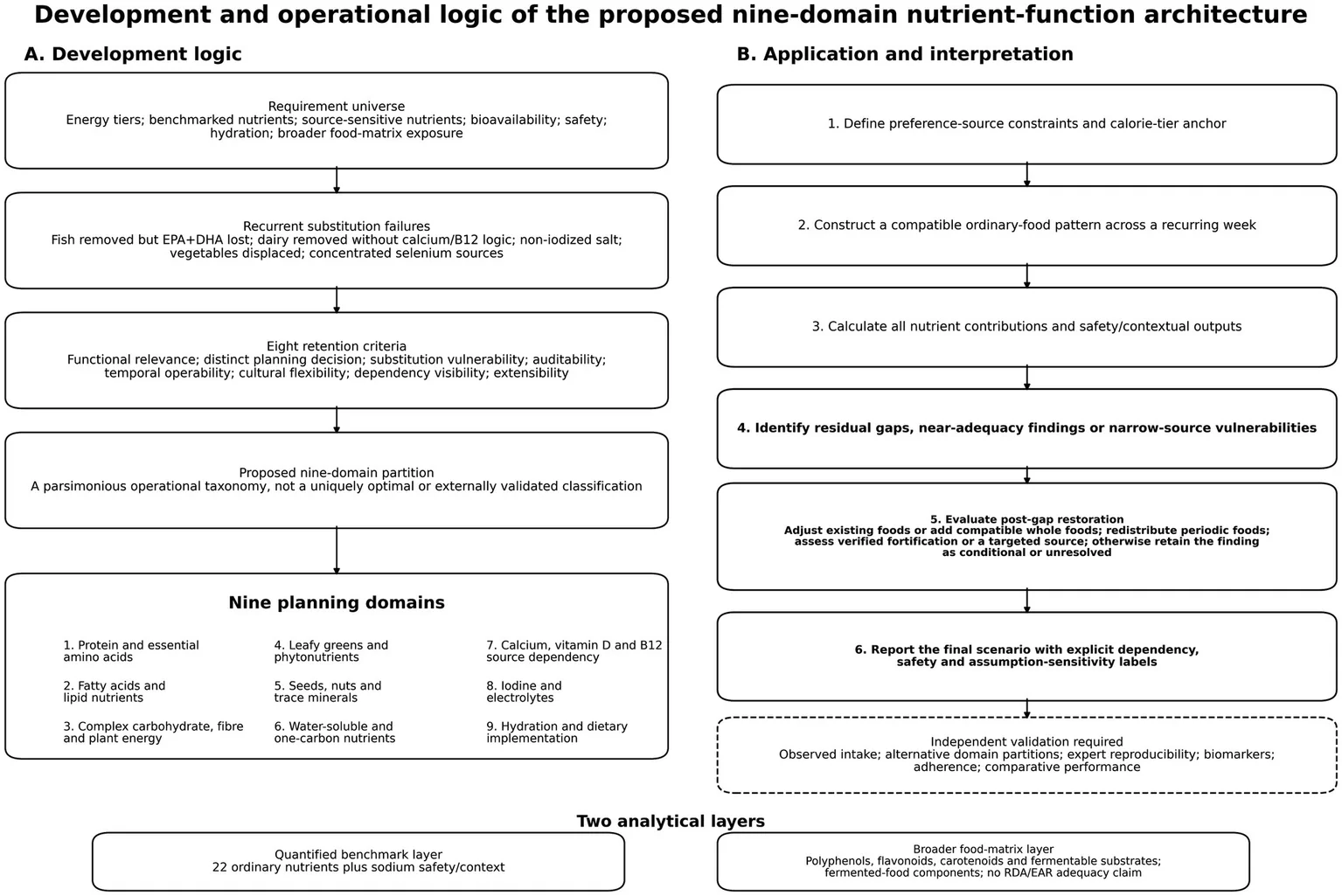
Development and operational logic of the proposed nine-domain nutrient-function architecture. Panel **(A)** presents the design sequence from the nutrient and operational requirement universe, through recurrent substitution failure modes and eight domain-retention criteria, to the proposed nine-domain partition. Panel **(B)** presents the application sequence: define preference-source constraints; construct compatible ordinary-food inputs; calculate nutrient outputs; identify residual gaps or narrow-source vulnerabilities; evaluate post-gap restoration options; and report conditionality and sensitivity. The nine domains preserve a quantified benchmark layer while retaining broader food-matrix exposure pathways that are not treated as RDA/EAR adequacy endpoints.

The third layer protects narrow-source, safety and implementation functions. Domain 7 identifies calcium, vitamin D and vitamin B12 source dependency and determines when ordinary compatible foods are insufficient. Domain 8 links iodine to a controlled source while interpreting sodium separately as a safety constraint. Domain 9 addresses hydration and practical distribution of the recurring pattern; fermented foods may occupy this domain where culturally compatible but are not assumed to be probiotic or mandatory for benchmark adequacy. Table 1 and Figure 1 summarise the domain decisions, quantified functions, broader food-matrix pathways and boundaries.

**Table 1:** Operational domains, quantified functions, broader food-matrix pathways and boundaries.

Domain	Primary planning decision	Quantified or flagged functions	Broader food-matrix pathway	Main boundary or dependency
1. Protein and essential amino acids	Select sufficient, preference-compatible protein sources and preserve quality logic.	Protein; source-linked iron, zinc, B12, B6, niacin and selenium are counted globally.	Choline and source-dependent creatine, carnitine, taurine or bioactive peptides.	A protein replacement may not restore all co-delivered nutrients.
2. Fatty acids and lipid nutrients	Separate general fat provision from ALA and preformed EPA + DHA decisions.	ALA; EPA + DHA; vitamin D, B12, iodine and selenium where supplied by fish.	Seed phenolics, lignans, phytosterols and fat-assisted carotenoid absorption.	Fish-free patterns require a separate EPA + DHA decision; algal sources do not reproduce all fish nutrients.
3. Complex carbohydrate, fibre and plant energy	Provide scalable plant energy, fibre, potassium, magnesium and food volume.	Fibre, magnesium, potassium, B vitamins and source-dependent vitamin C/carotenoids.	Resistant starch, beta-glucans, arabinoxylans, oligosaccharides, phenolic acids and saponins.	Lower energy tiers require greater nutrient density; not all fibre is prebiotic.
4. Leafy greens and phytonutrients	Protect high-density vegetable exposure from displacement by energy- or protein-dense foods.	Folate, vitamin K, carotenoid-derived vitamin A, vitamin C, magnesium, potassium and some iron.	Flavonoids, carotenoids, glucosinolates where crucifers are used, and diverse phenolics.	Broader phytonutrient exposure is not an RDA/EAR adequacy endpoint.
5. Seeds, nuts and trace minerals	Use nutrient-dense seeds/nuts while controlling concentrated trace-mineral sources.	Vitamin E, magnesium, zinc, selenium, ALA and protein contributions.	Lignans, phytosterols, tocopherol forms and seed/nut polyphenols.	Selenium sources require dose and upper-limit control.
6. Water-soluble and one-carbon nutrients	Make B-vitamin, folate and choline-related source logic explicit.	B1, B2, B3, B6, folate; source-specific B12 contributions counted globally.	Choline, inositol and source-dependent cofactors not fully benchmarked in the current model.	B12 is principally reviewed in Domain 7 to avoid double responsibility.
7. Calcium, vitamin D and vitamin B12 source dependency	Identify whether compatible foods supply these narrow-source nutrients and expose residual dependency.	Calcium, vitamin D and vitamin B12; riboflavin and protein co-delivery.	Fermented-dairy peptides or live-culture exposure where product-specific evidence supports it.	Fortification is a post-gap restoration option, not a domain-development assumption; label verification is required.
8. Iodine and electrolytes	Preserve a controlled iodine source while keeping sodium safety separate.	Iodine; sodium safety/context; potassium context where relevant.	No independent broader-bioactive claim is assigned to this domain.	Generic salt does not establish iodine adequacy; sodium is not an ordinary adequacy endpoint.
9. Hydration and dietary implementation	Ensure water provision and practical distribution of the recurring pattern.	Hydration is operational; no independent RDA-style adequacy score is assigned in the current model.	Fermented-food exposure, organic acids, possible live cultures and microbiota-supportive food structure.	Fermented foods are not automatically probiotic; microbial metabolites are not automatically postbiotics.

Domains are nutrient-function checkpoints, not rigid food groups or replacements for conventional nutrient categories. The nine-domain structure is proposed here as a planning taxonomy for scrutiny, refinement and future external evaluation. Domains were held constant while food sources and quantities changed by dietary preference and calorie tier.

### 2.5 Quantified benchmark layer and broader food-matrix layer

The model used two analytically distinct layers. The quantified layer calculated energy and 23 non-energy outputs. After sodium was treated as a safety/contextual nutrient, 22 nutrients remained in ordinary adequacy summaries. Vitamin D remained within the calculated set but was assigned a conditional interpretation because dietary intake alone cannot establish biological sufficiency.

The broader food-matrix layer documented pathways for carotenoids, phenolic acids, flavonoids, lignans, phytosterols, resistant starch, beta-glucans, arabinoxylans, oligosaccharides, fermentation products and selected source-dependent compounds such as choline, creatine, carnitine and taurine. These pathways were represented by the food structure but were not given adequacy scores. Not all fibre was classified as prebiotic; fermented foods were not automatically classified as probiotic; microbiota-derived metabolites were not labelled postbiotics; and adaptogenic botanicals were not part of the fixed model (25–27).

### 2.6 Dietary source-availability archetypes

Eleven variants were selected as source-availability archetypes rather than survey-derived prevalence categories or an exhaustive user taxonomy: (V01) Non-Vegetarian General; (V02) Red + White Meat including Beef and Pork; (V03) Red + White Meat excluding Beef and Pork; (V04) Only Red Meat; (V05) Only White Meat including Fish; (V06) Only White Meat excluding Fish; (V07) Only Fish/Pescatarian; (V08) Lacto-Ovo Vegetarian/Egg Vegetarian; (V09) Ovo-Vegetarian; (V10) Lacto-Vegetarian/Pure Vegetarian; and (V11) Vegan.

The set was chosen to expose distinct source-loss problems. V01-V03 represented broad animal-source availability and culturally relevant red-meat boundaries. V04-V06 separated red meat, white meat and fish availability to isolate preformed EPA + DHA and co-nutrient consequences. V07 tested a fish-centred animal-food pattern. V08-V10 separated egg and dairy availability within vegetarian patterns. V11 removed all animal-derived foods and therefore provided the most restrictive stress test for vitamin B12, calcium, vitamin D and EPA + DHA source restoration. The variants were not ranked and did not imply that every individual must fit a single fixed category; a real pattern can be mapped to the relevant source constraints.

### 2.7 Calorie-tier anchors and selective scaling

Four maintenance calorie-tier anchors were used: Lite, 1,500 kcal/day; Standard, 2,000 kcal/day; Active, 2,350 kcal/day; and High-Output, 2,800 kcal/day. These were modelling anchors selected to examine the architecture under lower-volume, central-reference, active and upper-scaling conditions. They were not individualized energy prescriptions or population prevalence classes.

Scaling was selective rather than proportional across all foods. Energy-bearing foods such as grains, legumes, roots, protein portions, dairy or plant-milk equivalents and culinary fats were adjusted within the tier structure. Concentrated or safety-sensitive sources, including iodized salt, Brazil nut or selenium equivalents and targeted EPA + DHA, were capped or independently specified rather than multiplied linearly with energy. Leafy greens and other nutrient-dense foods were protected from being reduced solely to meet a calorie target. Table 2 summarises the variant and tier logic.

**Table 2:** Selection logic for dietary preference archetypes, calorie tiers and internal model-evaluation scenarios.

Model element	Categories or anchors	Selection logic	Interpretation
Architecture	Nine fixed planning domains	Derived from requirement coverage, substitution failures and eight retention criteria.	Proposed operational partition; not claimed as uniquely optimal or externally validated.
Animal-source breadth variants	V01-V03: general omnivorous; red + white meat with or without beef/pork	Tests broad source availability and culturally/religiously relevant exclusions.	Source archetypes, not prevalence categories.
Meat-restricted variants	V04-V06: red-only; white meat with fish; white meat without fish	Separates red/white meat and fish availability to expose EPA + DHA and co-nutrient substitutions.	Fish exclusion requires a separate preformed EPA + DHA decision.
Fish-focused variant	V07: pescatarian	Tests fish/seafood availability with exclusion of terrestrial meat.	Egg/dairy availability may still vary within practical implementation.
Vegetarian variants	V08-V10: lacto-ovo, ovo, lacto/pure vegetarian	Separates egg and dairy availability and their effects on protein, calcium and B12 pathways.	Final adequacy may depend on post-gap restoration sources.
Plant-only variant	V11: vegan	Tests complete exclusion of animal-derived foods.	Final outputs are conditional where fortified or targeted sources are required.
Calorie tiers	Lite 1,500; Standard 2,000; Active 2,350; High-Output 2,800 kcal/day	Tests selective scaling across lower to higher maintenance-energy contexts.	Anchors are not individualized prescriptions.
Scenario structure	11 variants x 4 tiers = 44 scenarios	Applies identical domain rules under contrasting source and energy constraints.	Internal model evaluation, not independent validation.

Energy was used as a calorie-tier anchor and excluded from non-energy adequacy counts; sodium was retained as a safety/contextual row, leaving 22 ordinary adequacy nutrients per scenario.

### 2.8 Recurring-week unit and meal distribution

The modelling unit was the recurring dietary pattern expressed as average daily food equivalents. Foods used daily were entered directly. Foods used several times per week were multiplied by their weekly frequency and divided by seven. This allowed fish, meats, eggs, legumes, vegetables and other rotating foods to contribute to a comparable daily nutrient total without implying identical consumption every day.

The model evaluated the complete recurring pattern rather than the adequacy of each meal. The same food-equivalent pattern could be distributed across one, two or more meals according to practical preference. Meal frequency, postprandial response and temporal protein distribution were not independent outcomes in this study.

### 2.9 Iterative food-equivalent formulation

Food quantities were developed through iterative constraint-based formulation rather than by applying a single fixed serving to all variants. Candidate quantities were adjusted against six prespecified checks: (1) the scenario had to remain within its calorie-tier band; (2) all nine planning domains had to remain represented or explicitly flagged; (3) ordinary nutrient adequacy had to be calculated against the declared benchmarks; (4) narrow-source or fortification dependencies had to remain visible; (5) upper-limit and sodium screens had to be reviewed; and (6) quantities had to remain interpretable as practical food or household equivalents.

The adjustment sequence began with permitted foods consistent with the relevant source archetype. Quantities and combinations within those foods were revised first. Where a nutrient function remained vulnerable, another compatible whole-food source was considered. Periodic foods could then be redistributed within the recurring week. Only after these food-based options were evaluated were verified fortified foods or defined targeted sources considered. The process stopped when the final pattern was within the tier band, contained no concealed moderate or major ordinary gap, retained unresolved findings as near or conditional, and disclosed all material dependencies and safety flags.

This was a target-pattern construction process, not a statistical estimate of an optimal diet and not proof that the final quantities are unique. Many alternative food combinations could satisfy similar constraints. The analysis evaluates whether the proposed architecture can produce auditable target patterns under the declared assumptions.

### 2.10 Post-gap restoration and fortification chronology

Fortification was not a criterion used to derive the domains and was not assumed in advance to make restrictive diets adequate. The analytical sequence was: define the permitted food-source universe; construct the recurring pattern through the nine domains; calculate intake; detect residual gaps or source vulnerabilities; evaluate restoration options; recalculate; and retain the final output as intrinsic, restored, conditional or unresolved.

The restoration hierarchy was: revise quantities or combinations within existing foods; introduce another culturally and preference-compatible whole food; redistribute periodic foods; evaluate a verified fortified food; evaluate a defined targeted source such as algal EPA + DHA; or retain a conditional/unresolved finding. Adequacy dependent on fortification remained conditional on product composition, availability and consumption. A targeted source was not assumed to reproduce the full nutrient package of the excluded food.

The public supplement preserves the final evaluated scenarios and defined sensitivity reversals rather than a complete numerical archive of every intermediate formulation. The manuscript therefore describes the documented modelling sequence but does not present unretained intermediate food-only values as measured results.

### 2.11 Food-composition and benchmark sources

Food-composition values were drawn principally from Indian Food Composition Tables and mapped to the foods or representative food equivalents used in the scenarios (14). Where an exact item was unavailable, a documented representative composite, verified product label or secondary composition source was used and flagged as assumption-dependent. Fortified products were counted only when their nutrient specifications were declared. Food composition was interpreted as estimated rather than fixed because cultivar, geography, processing, cooking, edible portion and brand can materially alter values.

The conservative benchmark set used the higher adult male or female value where sex-specific values differed. Sodium was treated as a safety limit rather than an adequacy target. EPA + DHA used a contextual adult target because an ICMR RDA/EAR was not identified. Upper-limit values were used as screening thresholds and interpreted according to nutrient form, because several ULs apply primarily to preformed, synthetic, fortified or supplemental forms rather than total food-derived equivalents.

### 2.12 Nutrients evaluated

Energy was the calorie-tier anchor. The 23 non-energy outputs were protein; dietary fibre; calcium; iron; zinc; magnesium; potassium; sodium; selenium; iodine; vitamins A, B1, B2, B3, B6, folate, B12, C, D, E and K; ALA; and EPA + DHA. Sodium was excluded from ordinary adequacy counts. Vitamin D was calculated but interpreted separately as conditional. The ordinary adequacy denominator was therefore 22 nutrients per scenario.

### 2.13 Intake, adequacy and upper-limit calculations

For scenario s and nutrient n, modelled intake was calculated as I(s,n) = sum over foods f of q(s,f) x c(f,n), where q(s,f) is the average daily quantity or converted food-equivalent amount and c(f,n) is the nutrient composition per calculation unit. All nutrients from a food were included regardless of its primary domain assignment.

Adequacy was calculated as A(s,n) (%) = 100 x I(s,n)/B(n), where B(n) is the selected benchmark. Energy was excluded from non-energy counts, and sodium was excluded from ordinary adequacy summaries. Where an applicable upper limit was available, UL exposure was calculated as 100 x I(s,n)/UL(n).

### 2.14 Adequacy, dependency and safety classifications

Ordinary nutrients at or above 100% of benchmark were classified as meeting benchmark; 80–99% as near adequacy/watch; 50–79% as a moderate gap; and below 50% as a major gap. Vitamin D was classified as conditional regardless of dietary percentage because serum sufficiency depends on diet, sun exposure, season, geography, skin pigmentation, absorption and supplementation (18). Iron, zinc and calcium carried bioavailability cautions where plant, leafy or fortified sources dominated (30).

Dependency flags distinguished natural/base-food supply, verified fortified-source dependency, targeted-source dependency, controlled-source dependency, conditionality, bioavailability caution and safety sensitivity. Upper-limit screening classified rows as no applicable UL, within UL, UL watch at 80–99% or exceeds UL at 100% or more. These categories were computational review flags, not diagnoses of toxicity.

### 2.15 Sensitivity analyses

Ten sensitivity families tested the stability and interpretation of the final scenarios: fortified plant milk replaced by non-fortified plant milk; fortified nutritional yeast replaced by non-fortified yeast; fish removed and replaced only by algal EPA + DHA; algal EPA + DHA removed from fish-free variants; dairy calcium replaced with a fortified plant source; a capped selenium source removed or doubled; iodized salt replaced with non-iodized salt; Lite compared with Standard tier; root/tuber replaced by calorie-equivalent grain in selected culturally restricted scenarios; and iron/zinc adequacy interpreted through conservative bioavailability-adjusted proxies.

A row was classified as highly sensitive when the absolute change was at least 25 percentage points or the alternative output became a moderate or major gap; moderately sensitive when the change was 10–24.9 percentage points; and low sensitivity otherwise. Scenario-level classes summarised the number of highly sensitive rows. These thresholds describe only the specified perturbations and do not imply universal robustness.

### 2.16 Supplementary calculation trail

Supplementary data sheet 1 documents the architecture-development sequence, domain-to-nutrient map, broader food-matrix pathways, variant and tier rationale, post-gap restoration sequence, food-source rows, composition assumptions, benchmark table, scenario-energy checks, intake calculations, adequacy categories, dependency flags, safety screens, scenario and variant summaries, nutrient-risk outputs and sensitivity tests. The workbook is intended to permit recalculation and scrutiny of the final modelled scenarios.

### 2.17 Scope exclusions

The analysis did not use observed dietary records, national survey microdata, biomarkers, clinical outcomes, adherence, acceptability, cost, cooking feasibility, medication data or longitudinal follow-up. It did not estimate nutrient absorption, microbiome composition, postprandial response or disease risk. It was not designed for pregnancy, lactation, childhood, adolescence, chronic kidney disease, advanced liver disease, severe malabsorption, active inflammatory bowel disease, eating disorders or other contexts requiring individualized medical nutrition therapy. The outputs are target-pattern modelling estimates, not evidence of universal individual sufficiency.

## 3 Results

### 3.1 Scenario coverage and calculation completeness

The model generated 44 complete internal model-evaluation scenarios from 11 dietary source archetypes and four calorie-tier anchors. Every scenario retained the nine planning domains while permitted foods and quantities changed according to preference and energy tier. The four anchors were 1,500, 2,000, 2,350 and 2,800 kcal/day.

Energy was used as the tier anchor and excluded from non-energy nutrient rows. The model generated 23 non-energy rows per scenario, or 1,012 scenario-nutrient rows. Sodium remained a safety/contextual row; ordinary adequacy summaries therefore used 22 nutrients per scenario and 968 rows. The final scenarios were available for adequacy, dependency, safety and sensitivity analysis.

### 3.2 Operationalization of the nine domains

All 44 scenarios could be expressed through the nine-domain structure. Food exclusions triggered a check of the affected nutrient-function pathway rather than a simple deletion. Fish-containing scenarios used fish for preformed EPA + DHA and co-delivered nutrients, while fish-free scenarios required an independent EPA + DHA decision. Dairy-free patterns required explicit calcium and vitamin B12 source review. Plant-heavy patterns preserved legumes, grains, leafy greens, nuts and seeds rather than increasing protein foods at the expense of fibre and vegetable functions. Iodine was linked to iodized salt or another controlled source rather than generic salt.

The architecture also retained food positions relevant to broader, non-benchmarked exposure. Whole grains, legumes, roots, leafy and non-starchy vegetables, fruits, nuts, seeds and fermented foods occurred across the scenarios even though their individual polyphenols, flavonoids, prebiotic substrates or fermentation products were not assigned adequacy scores. Representative Standard Maintenance inputs for mixed, lacto-vegetarian and vegan scenarios are shown in Table 3.

**Table 3:** Representative standard maintenance final evaluated inputs, dependencies and outputs.

Nine-domain input	V01 Non-vegetarian general	V10 lacto-vegetarian/pure vegetarian	V11 vegan
Protein and essential amino acid domain	2 eggs daily + 105 g cooked lean red meat on 4 days/week + 95 g cooked poultry/white meat on 3 days/week	150 g low-fat paneer/strained curd protein equivalent daily	220 g cooked soy/tempeh/soy-chunk equivalent + 20 g verified pea/soy protein equivalent
Fatty acid and lipid-nutrient domain	150 g low-mercury fatty fish on 4 days/week + 12 g ground flax/chia daily + 15 g unsaturated culinary oil	Algal EPA/DHA 375 mg + 12 g ground flax/chia + 20 g unsaturated culinary oil	Algal EPA/DHA 375 mg + 15 g ground flax/chia + 20 g unsaturated culinary oil
Complex carbohydrate, fibre and plant-energy domain	200 g cooked whole grain/pseudocereal + 150 g cooked lentil/bean mix + 244 g cooked root/tuber/pumpkin/carrot equivalent	220 g cooked whole grain/pseudocereal + 275 g cooked lentil/bean mix + 300 g cooked root/tuber/pumpkin/carrot equivalent	220 g cooked whole grain/pseudocereal + 275 g cooked lentil/bean mix + 300 g cooked root/tuber/pumpkin/carrot equivalent
Leafy green and phytonutrient domain	250 g cooked leafy greens + 100 g vitamin-C-rich vegetable/fruit-equivalent	360 g cooked leafy greens + 100 g vitamin-C-rich vegetable/fruit-equivalent	380 g cooked leafy greens + 100 g vitamin-C-rich vegetable/fruit-equivalent
Seed, nut and trace-mineral domain	40 g iron-zinc seed blend	75 g iron-zinc seed blend + 1 Brazil nut on 3 days/week	85 g iron-zinc seed blend + 1 Brazil nut on 3 days/week
Water-soluble vitamin and one-carbon nutrient domain	12 g verified fortified nutritional yeast where required after gap assessment	15 g verified fortified nutritional yeast where required after gap assessment	20 g verified fortified nutritional yeast + 10 g soy lecithin granules as post-gap restoration inputs
Calcium, Vitamin D and Vitamin B12 source-dependency domain	400 mL curd/kefir/milk; use a verified fortified equivalent only if a residual source gap is identified	600 mL curd/kefir/milk; verified fortified equivalent only where required after gap assessment	750 mL verified fortified plant-milk equivalent used as a post-gap restoration source
Iodine and electrolyte domain	Controlled iodized salt or verified iodine source within total sodium limits	Controlled iodized salt or verified iodine source within total sodium limits	Controlled iodized salt or verified iodine source within total sodium limits
Hydration and dietary-implementation domain	2.5–3 L water; curd/kefir may provide fermented-food exposure, otherwise 50–75 g compatible fermented vegetables	2.5–3 L water; curd/kefir may provide fermented-food exposure, otherwise 50–75 g compatible fermented vegetables	2.5–3 L water + 50–75 g compatible fermented vegetables; no probiotic claim is assumed
Ordinary adequacy result	20/22 ordinary adequacy nutrients met; 1 near-adequacy/watch nutrient; 0 moderate or major ordinary gaps.	21/22 ordinary adequacy nutrients met; 0 near-adequacy/watch nutrients; 0 moderate or major ordinary gaps.	21/22 ordinary adequacy nutrients met; 0 near-adequacy/watch nutrients; 0 moderate or major ordinary gaps.
Conditional/watch nutrients	Vitamin D conditional (74.8% of benchmark); iron near adequacy/watch (95.7%) with bioavailability caution.	Vitamin D conditional (22.0% of benchmark); iron and zinc met benchmark but retain bioavailability caution.	Vitamin D conditional (50.0% of benchmark); iron and zinc met benchmark but retain bioavailability caution; B12 and calcium are label-dependent.
Safety/source-form interpretation flags	Sodium flagged as safety/contextual rather than ordinary adequacy; vitamin A, niacin and folate require source-form review; selenium within UL in this scenario.	Sodium flagged as safety/contextual; calcium at UL-watch; vitamin A, niacin and folate require source-form review.	Sodium flagged as safety/contextual; calcium at UL-watch; iron, vitamin A, niacin and folate require source-form review; selenium within UL.
Dependency and assumption-sensitivity interpretation	Fish and dairy provide several source pathways; final result showed high sensitivity to tested assumptions.	Fish-free adequacy uses algal EPA + DHA; any fortified source remains a post-gap dependency; final result showed high sensitivity.	Final adequacy is conditional on verified plant milk, fortified nutritional yeast, algal EPA + DHA and controlled iodine; high sensitivity.

Values are final evaluated average daily equivalents for three representative Standard Maintenance scenarios, not archived initial food-only iterations. Fortified or targeted sources shown in the table were used as post-gap restoration inputs and must be interpreted as conditional on verified composition and use. Ordinary adequacy results use the 22-nutrient denominator; vitamin D is reported separately as conditional. Full nutrient-level outputs for all four tiers and 11 variants are provided in Supplementary data sheet 1. These inputs are not individualized clinical prescriptions.

### 3.3 Overall ordinary nutrient adequacy

Across the 44 final evaluated scenarios, the mean number of ordinary nutrients meeting benchmark was 20.64 of 22. Scenario-level counts ranged from 19 to 21. No ordinary nutrient generated a moderate or major gap in the final scenarios. Values below full benchmark remained visible as near-adequacy findings rather than being adjusted or reclassified as adequate.

The denominator requires careful interpretation. Sodium was excluded because it was a safety limit rather than an adequacy target. Vitamin D remained among the 22 calculated nutrients but was classified as conditional rather than ordinary adequacy. Consequently, a scenario meeting 21 of 22 ordinary benchmarks could still have modelled vitamin D intake below, near or above 100% while remaining biologically conditional.

### 3.4 Variant-level findings

Variant means ranged from 20.25 to 21.00 of 22 ordinary nutrients. Non-Vegetarian General, both red-and-white-meat variants and Only Red Meat each averaged 20.25. White Meat including Fish, White Meat excluding Fish, Lacto-Ovo Vegetarian and Ovo-Vegetarian each averaged 20.75. Pescatarian, Lacto-Vegetarian and Vegan scenarios each averaged 21.00.

These values do not indicate that the vegan or vegetarian labels were intrinsically more adequate. They describe final evaluated target patterns after the architecture had identified and retained their required source pathways. The final vegan pattern depended on verified fortified sources for selected calcium and vitamin B12 inputs and on targeted algal EPA + DHA. Likewise, fish-free animal-food and vegetarian variants depended on algal EPA + DHA, while ovo-vegetarian and vegan patterns were sensitive to plant-milk fortification. Table 4 presents variant-level counts and principal dependency interpretations.

**Table 4:** Variant-level ordinary adequacy and principal source-dependency interpretation.

Variant archetype	Mean ordinary nutrients meeting benchmark (of 22)	Scenario range	Principal interpretation
V01 non-vegetarian general	20.25	19–21	Fish contributes EPA + DHA and co-nutrients; lower tiers retained iron/zinc watches.
V02 Red + White meat including beef and pork	20.25	19–21	Broad animal-source availability; lower tiers retained iron/zinc watches.
V03 Red + White meat excluding beef and pork	20.25	19–21	Culturally bounded mixed pattern; lower tiers retained iron/zinc watches.
V04 Only red meat	20.25	19–21	Fish absent; preformed EPA + DHA depends on targeted algal source; vitamin D remained low.
V05 Only white meat including fish	20.75	20–21	Fish retained; zinc watch occurred in one Active scenario.
V06 Only white meat excluding fish	20.75	20–21	Fish absent; EPA + DHA depends on algal source; zinc watch occurred in Lite.
V07 Only Fish/Pescatarian	21.00	21–21	Fish supplies EPA + DHA and co-nutrients; vitamin D remained conditional.
V08 Lacto-ovo vegetarian/Egg vegetarian	20.75	20–21	EPA + DHA depends on algal source; zinc watch occurred in Lite.
V09 ovo-vegetarian	20.75	20–21	Dairy absent; calcium/B12 interpretation sensitive to verified plant-milk fortification; zinc watch in Lite.
V10 Lacto-vegetarian/Pure vegetarian	21.00	21–21	EPA + DHA depends on algal source; vitamin D remained the lowest conditional nutrient.
V11 vegan	21.00	21–21	Final adequacy conditional on verified fortified calcium/B12 pathways and targeted algal EPA + DHA.

Values describe final evaluated target patterns after identified restoration steps. They do not establish adequacy of real-world diets by label alone. Vitamin D remained conditional in every variant.

### 3.5 Adequacy by calorie tier

Mean ordinary benchmark coverage increased from 20.00 nutrients in Lite Maintenance to 20.64 in Standard, 20.91 in Active and 21.00 in High-Output Maintenance. The corresponding mean near-adequacy counts were 1.00, 0.36, 0.09 and 0.00. Lite scenarios ranged from 19 to 21 nutrients meeting benchmark; Standard from 20 to 21; Active from 20 to 21; and all High-Output scenarios met 21.

**Figure 2:**
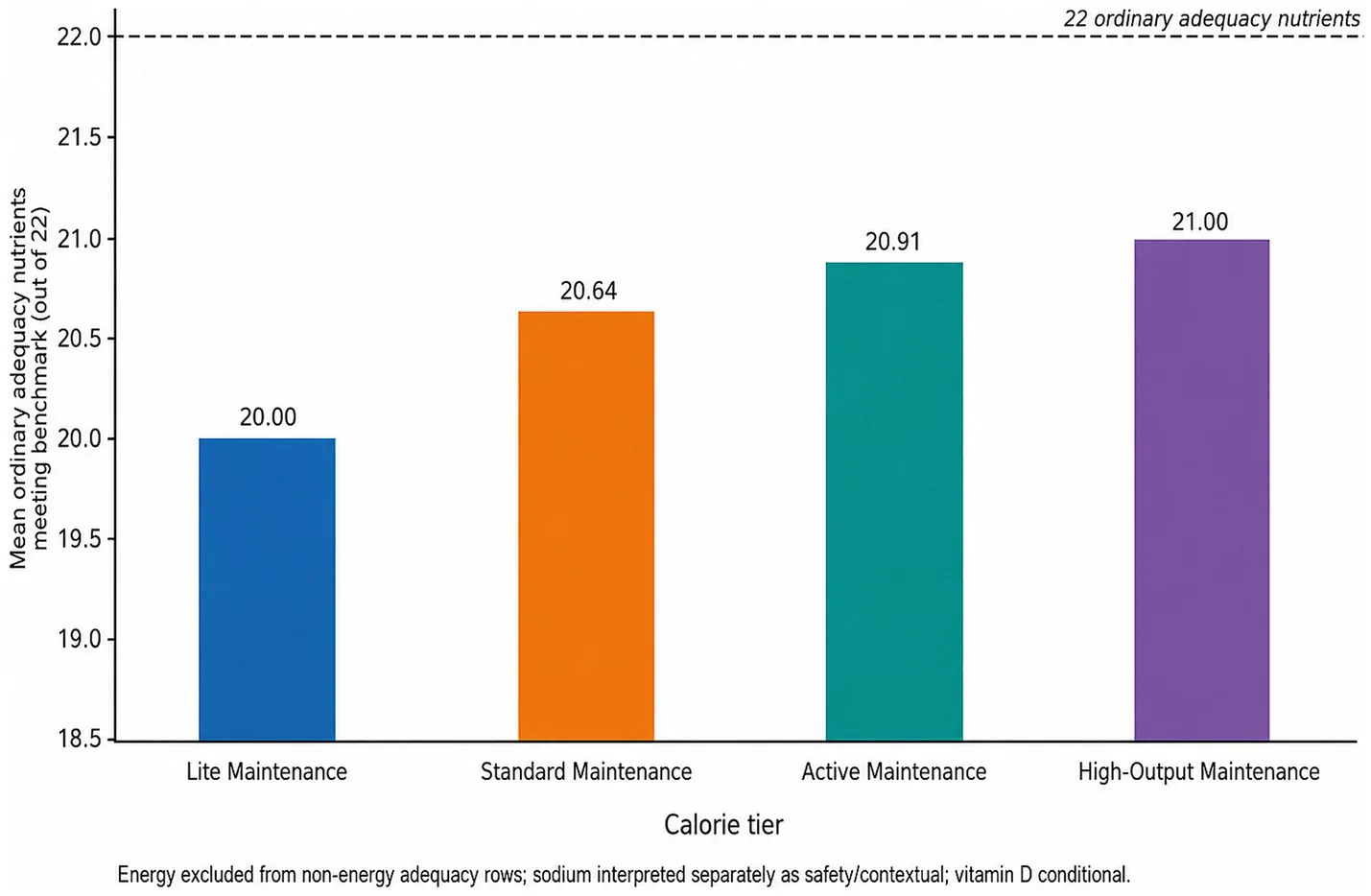
Mean number of benchmarked nutrients meeting adequacy by calorie tier. Mean number of ordinary adequacy nutrients meeting benchmark across the four calorie tiers. Energy was included in the model as a calorie-tier anchor but excluded from non-energy nutrient rows. Sodium was retained for safety/contextual interpretation and excluded from ordinary adequacy counts. The mean number of ordinary adequacy nutrients meeting benchmark increased from 20.00 of 22 nutrients in Lite Maintenance scenarios to 21.00 of 22 nutrients in High-Output Maintenance scenarios.

The gradient reflected additional nutrient margin at higher food volumes rather than a recommendation for higher energy intake. Lower-volume scenarios were more dependent on nutrient-dense food selection and were more likely to place iron or zinc near the benchmark. Figure 2 summarises the tier means.

### 3.6 Nutrient-level findings

**Figure 3:**
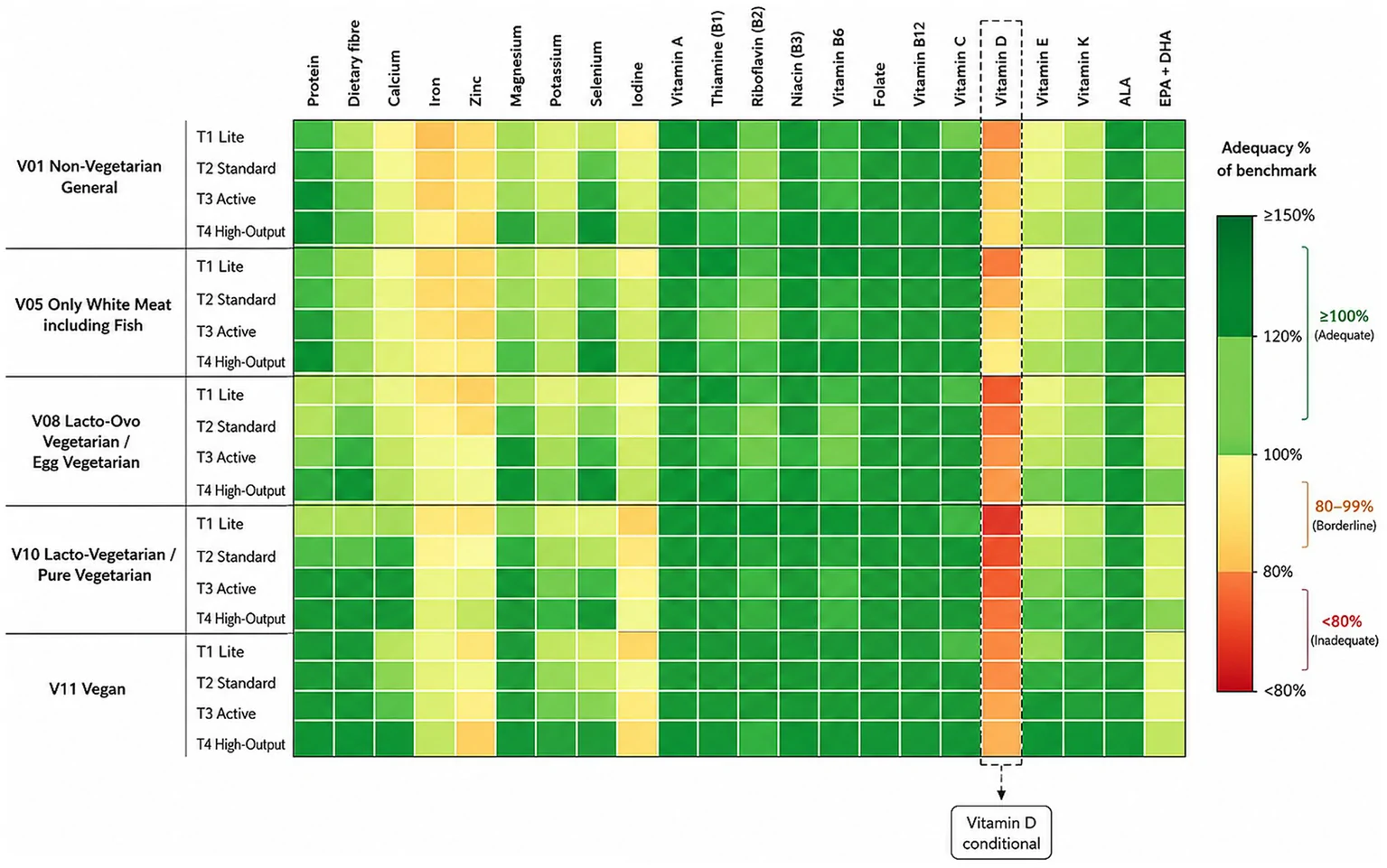
Representative nutrient adequacy heatmap across selected dietary variants and calorie tiers. Representative heatmap of conservative adequacy percentages across selected dietary variants and calorie tiers. The main heatmap displays representative variants rather than all 44 modelled scenarios to preserve readability; full scenario-level outputs are provided in the supplementary dataset. Energy was excluded because it was a calorie-tier anchor. Sodium is interpreted in the safety/contextual layer rather than as an ordinary adequacy endpoint, and vitamin D is interpreted as conditional.

Twenty-one of the 22 ordinary nutrients met their benchmark in every scenario or remained above the benchmark apart from isolated near-adequacy findings. Iron produced eight near-adequacy findings and met benchmark in 36 scenarios; its lowest value was 87.94% in the Only Red Meat Lite scenario. Zinc also produced eight near-adequacy findings and met benchmark in 36 scenarios; its lowest value was 88.15% in the Ovo-Vegetarian Lite scenario. The iron watches occurred primarily in the lower-tier broad-meat scenarios, whereas zinc watches occurred across selected lower-tier and plant-heavy patterns (Figure 3).

Protein, fibre, calcium, magnesium, potassium, selenium, iodine, vitamins A, B1, B2, B3, B6, folate, B12, C, E and K, ALA and EPA + DHA met their selected benchmark in all final scenarios. This statement concerns calculated intake and does not remove source-dependency, bioavailability or safety qualifications. For example, calcium and vitamin B12 could meet benchmark only because verified source assumptions were retained in selected variants; iron and zinc totals did not establish absorbed amounts; and EPA + DHA adequacy in fish-free patterns depended on a targeted algal source.

Vitamin D was conditional in all 44 scenarios. Modelled dietary percentages ranged from 18.0% in Lacto-Vegetarian Lite to above 100% in some fish-containing High-Output scenarios, but dietary percentage was not treated as proof of vitamin D sufficiency because serum status also depends on sun exposure, geography, season, skin pigmentation, absorption and supplementation (18).

### 3.7 Restoration dependencies and sensitivity findings

Sensitivity tests confirmed that several final outputs depended materially on restoration or controlled-source assumptions. Removing plant-milk fortification affected calcium, vitamin D and vitamin B12 across eight ovo-vegetarian and vegan scenarios; 10 of 24 tested rows fell below 80% of benchmark. Treating nutritional yeast as non-fortified affected six B-vitamin/B12 outputs across all 44 scenarios; 30 of 264 rows fell below 80%. Removing algal EPA + DHA from the 24 fish-free scenarios moved EPA + DHA below 80% in every case.

Replacing fish with algal EPA + DHA preserved the defined EPA + DHA target but altered vitamin D, vitamin B12, selenium and iodine co-delivery; 18 of 100 tested rows moved below 80%. Replacing dairy with a fortified plant source changed calcium, vitamin D and vitamin B12 margins, showing that same-domain substitution did not imply identical nutrient composition. Replacing iodized salt with non-iodized salt moved iodine below 80% in 14 scenarios. Removing the capped selenium source produced three below-80% selenium rows, whereas doubling it increased excess-risk concern rather than improving interpretation.

**Figure 4:**
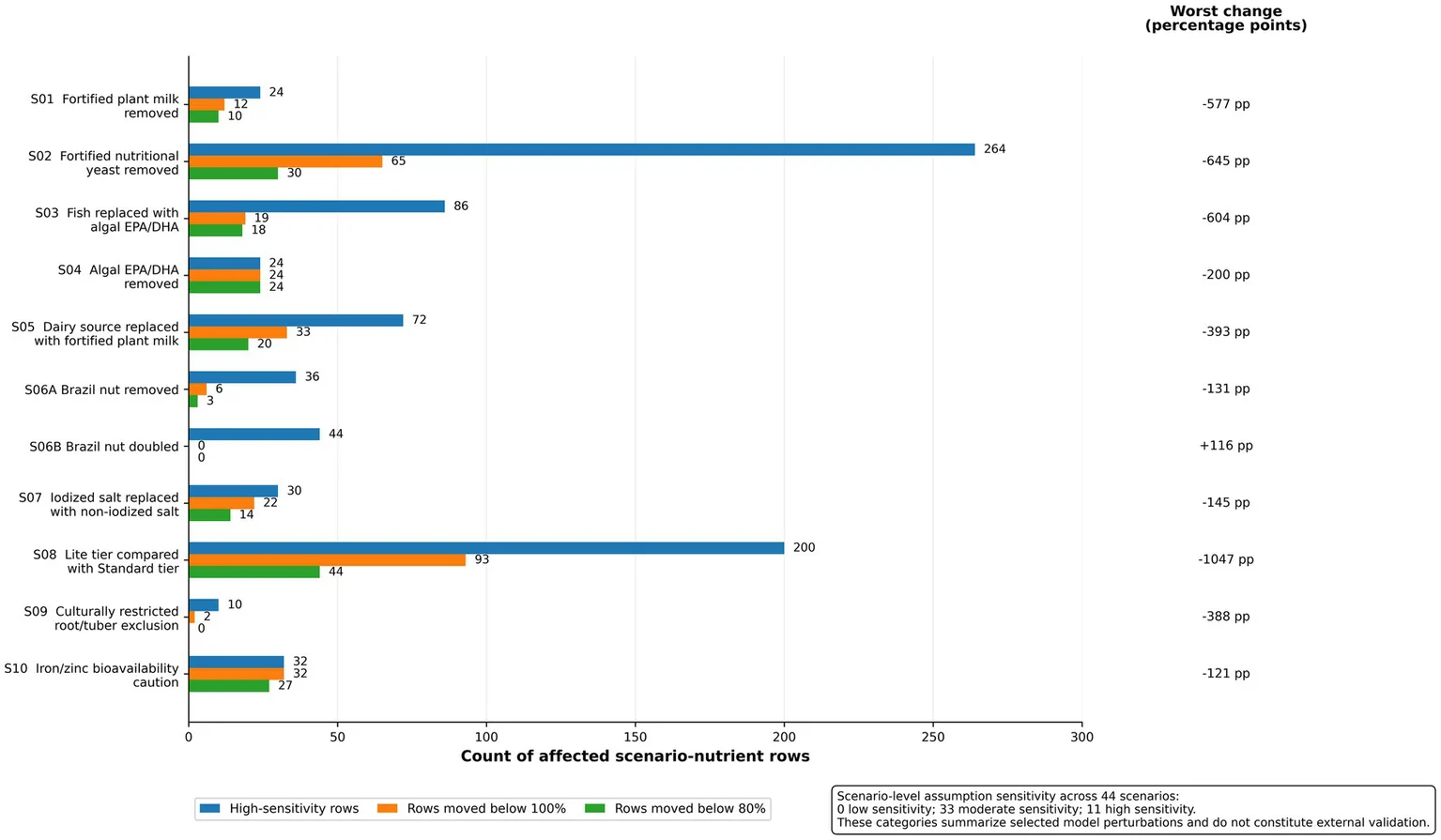
Sensitivity of final modelled nutrient adequacy to post-gap restoration and controlled-source assumptions. Sensitivity tests assessed whether final modelled nutrient adequacy changed when post-gap restoration or controlled-source assumptions were altered. Tests included fortified versus non-fortified plant milk, fortified versus non-fortified nutritional yeast, fish-to-algal EPA + DHA substitution, algal EPA + DHA removal, dairy-to-fortified-plant calcium substitution, selenium-source removal or doubling, iodized versus non-iodized salt, Lite versus Standard calorie tier, culturally restricted root/tuber substitution, and iron/zinc bioavailability caution. The outputs identify assumption dependence within the model and are not estimates of real-world causal effects.

The conservative iron and zinc bioavailability test affected 16 plant-heavy scenarios and moved 27 of 32 rows below 80%, illustrating the difference between total calculated intake and an absorption-sensitive interpretation. Root/tuber replacement by calorie-equivalent grain in selected culturally restricted patterns changed fibre, potassium, vitamin A, vitamin C and magnesium margins but did not produce a below-80% result in the tested rows. Figure 4 summarises the principal perturbations.

### 3.8 Safety and upper-limit screening

Across the full 1,056 scenario-output safety-screen rows, including the 44 energy-anchor rows, 484 had no applicable upper-limit comparison, 374 were within the selected threshold, 47 were classified as UL watch and 151 exceeded the selected threshold. The 151 exceedance flags were concentrated in niacin (44 rows), vitamin A (43), folate (40), sodium (18), calcium (3) and iron (3). The 47 watch flags comprised sodium (26), iron (12), calcium (4), folate (4) and vitamin A (1).

These counts were not interpreted as 151 toxic exposures. The benchmark table intentionally uses conservative screening values, but several ULs are source-form specific. The vitamin A UL principally concerns preformed retinol rather than total carotenoid-derived RAE; the niacin UL principally concerns supplemental or fortified nicotinic acid/niacinamide; and the folate UL concerns synthetic folic acid rather than natural food folate. Sodium, calcium and iron require dose and clinical-context review. Accordingly, the safety output identifies rows requiring source-form adjudication before individual application.

### 3.9 Scenario-level assumption sensitivity

No scenario met the low-sensitivity criterion across all tested perturbations. Thirty-three scenarios were classified as moderately sensitive and 11 as highly sensitive. High-sensitivity classification was concentrated in the 11 Standard-tier reference scenarios because several cross-tier and source-replacement tests used Standard Maintenance as the comparison baseline. The classification therefore reflects both model dependence and sensitivity-test design, not an intrinsic ranking of the dietary variants.

The sensitivity profile shows that broad base-case adequacy was maintained only while the declared food-source, fortification, controlled iodine, selenium and calorie-tier assumptions were preserved. It defines the conditions under which the final scenarios apply and does not independently validate the architecture.

## 4 Discussion

### 4.1 Principal findings

This study converted an author-developed dietary framework into a transparent nutrient-function modelling method and applied it across 44 internally constructed preference-tier scenarios. The final scenarios met a mean of 20.64 of 22 ordinary nutrient benchmarks, with no moderate or major ordinary gaps. The scenario range of 19–21 and the improvement from Lite to High-Output tiers show that the architecture was able to preserve broad benchmark coverage across source restrictions while lower food-volume patterns retained less margin.

The adequacy count is only one part of the result. Vitamin D remained conditional in all scenarios; iron and zinc generated the only ordinary near-adequacy findings; selected vegetarian, vegan, dairy-free and fish-free scenarios depended on verified fortified or targeted sources; and the safety layer showed that total-intake comparisons can be misleading unless nutrient form is considered. The architecture therefore did not simply generate an adequacy score. It exposed the assumptions and compensatory sources required to produce that score.

### 4.2 From dietary assessment to dietary construction

Food-based dietary guidelines, diet-quality indices and intake-assessment tools answer related but different questions. Guidelines translate nutrient science into population-facing food advice; indices evaluate adherence, adequacy, moderation or balance; and dietary records or digital systems estimate what was consumed (9–11). The proposed architecture addresses the preceding construction decision: when a target pattern is being created or adapted, what nutrient functions must remain visible so that a preference-based substitution does not silently remove them?

This distinction is illustrated by fish, dairy and iodized salt. A fish-free pattern is not nutritionally defined only by the absence of fish; it requires a separate decision about preformed EPA + DHA and acknowledgement that algal EPA + DHA does not reproduce vitamin D, vitamin B12, iodine and selenium co-delivery. A dairy-free pattern requires a calcium-B12-vitamin D source analysis rather than a beverage replacement based on appearance or use. A salt substitution requires verification of iodine while simultaneously controlling sodium. These are construction decisions rather than diet labels.

The method is therefore complementary to existing approaches rather than a claim of superiority. Its potential value lies in connecting source-level construction, quantitative benchmark comparison, dependency flags, safety screens and sensitivity tests in one auditable workflow.

### 4.3 Scientific interpretation of the nine-domain architecture

The nine domains are planning checkpoints, not mutually exclusive nutrient classes. They were retained because each represented a decision that could fail under substitution, could be calculated or flagged and could be delivered through culturally variable foods. The number nine is not itself the scientific claim. The claim is that an explicit requirement universe, failure-mode map, retention criteria and boundary rules can make target-pattern construction more reproducible.

The architecture also prevents one dimension of a food from standing in for its full nutritional role. Protein equivalence does not guarantee fatty-acid or micronutrient equivalence; calorie equivalence does not guarantee fibre or phytonutrient equivalence; and a fortified product can restore selected nutrients without reproducing the complete food matrix of the excluded source. Because all nutrients were counted globally, the model could assign one primary planning role without discarding co-delivered nutrients.

The present derivation remains a hypothesis-generating conceptual method. It was not developed by independent expert consensus or empirical clustering. Construct validation should test whether other researchers identify the same failure modes, assign foods similarly and retain the same or alternative domain boundaries. Competing architectures with fewer or more domains should be evaluated rather than assuming that the current partition is final.

### 4.4 Preference archetypes and restoration rather than presumed adequacy

The variant results require careful interpretation. Pescatarian, lacto-vegetarian and vegan variants achieved the highest final mean count, but this does not imply that these dietary identities are automatically more adequate. These were deliberately formulated target patterns. Their final results include the food-source corrections, verified fortified products and targeted sources identified through the modelling process. The study therefore evaluates whether adequacy can be constructed under the specified conditions, not whether unplanned real-world diets with the same labels are adequate.

Fortification was an outcome of residual-gap analysis rather than a parameter used to create the domains. Where ordinary compatible foods remained insufficient or impractical, the model evaluated a verified fortified product as one restoration pathway. The sensitivity findings show why this chronology matters. Removing plant-milk fortification reduced calcium, vitamin D and vitamin B12 in ovo-vegetarian and vegan scenarios; removing fortified nutritional yeast substantially changed B-vitamin and B12 margins. Adequacy conditional on such products must therefore remain explicitly label-dependent.

Targeted algal EPA + DHA played a similar role in fish-free patterns. Its inclusion restored the preformed EPA + DHA target but did not make fish and algal oil nutritionally identical. This distinction prevents a single-nutrient correction from being mistaken for complete source equivalence.

### 4.5 Nutrient breadth, compact meal patterns and the broader food matrix

A central practical implication is that broad nutrient-function coverage does not require every food or domain to appear in every meal. The recurring-week approach permits fish, eggs, legumes, dairy, meat, vegetables, nuts and seeds to rotate while their average daily contribution remains auditable. A compact one- or two-meal daily pattern may therefore carry a wide range of nutrient functions when complementary sources are deliberately distributed across the week. The present study does not compare meal frequencies, but it demonstrates that meal count alone is not an adequate proxy for nutrient breadth.

The quantified results capture only part of that breadth. The nine domains preserve repeated exposure to whole grains, legumes, roots, vegetables, leafy greens, fruits, nuts, seeds and fermented foods. These categories can provide chemically diverse carotenoids, phenolic acids, flavonoids, lignans, phytosterols, resistant starches and fermentable substrates in addition to the vitamins and minerals that were formally calculated. This food-matrix layer is one reason the architecture protects plant-food positions even in animal-source-rich variants.

At the same time, this wider spectrum should not be presented as quantified adequacy. Polyphenol content varies widely, not all fibre meets the definition of a prebiotic, fermented foods are not synonymous with probiotics, and microbial metabolites are not automatically postbiotics (25–27). The appropriate scientific claim is that the architecture structurally preserves broad exposure pathways and can be expanded as validated composition and reference data become available.

### 4.6 Calorie tiers and nutrient density

The calorie-tier gradient was modest but consistent. Lower-volume scenarios retained broad coverage but generated more iron and zinc watches, whereas High-Output scenarios met 21 of 22 ordinary benchmarks in every variant. This finding is consistent with the greater difficulty of fitting numerous nutrient functions into a smaller energy envelope.

The tiers were not produced by multiplying every food by the same factor. Nutrient-dense vegetables and narrow-source foods were protected or capped, while energy-bearing foods were selectively adjusted. This selective scaling is important because indiscriminate reduction can remove the very foods needed to preserve micronutrient density, whereas indiscriminate expansion can create sodium, selenium or other safety problems. The Lite tier should therefore be interpreted as a higher-density formulation challenge, not as evidence that lower-energy needs are inherently deficient.

### 4.7 Safety interpretation and source-form adjudication

The upper-limit screen is both a strength and a caution. It prevented apparent adequacy from being interpreted without considering excess, but it also showed that a simple comparison of total food-derived equivalents with a generic UL can overstate risk. Most exceedance flags involved vitamin A, niacin and folate, for which international ULs are form-specific. Total carotenoid-derived vitamin A should not be equated with preformed retinol exposure; natural food niacin should not be treated identically to pharmacological or fortified niacin; and natural folate should not be treated identically to folic acid.

The safety output is therefore a triage layer. It identifies where the form, source, dose and duplication of a nutrient must be reviewed before individual application. Sodium requires particular attention because iodized salt simultaneously supplies iodine and contributes to sodium exposure. Calcium, iron and selenium also require context-sensitive interpretation. A future workbook should separate source forms more explicitly so that biologically inappropriate UL comparisons can be excluded rather than merely flagged.

### 4.8 Strengths

The study has several strengths. It evaluated every predefined variant across all four calorie tiers rather than presenting one illustrative diet. It used continuous gram or food-equivalent quantities and formal equations rather than relying only on household descriptions. It distinguished ordinary adequacy from conditional vitamin D, source dependency, bioavailability caution and safety review. It disclosed the post-gap role of fortification and targeted sources instead of presenting corrected patterns as naturally adequate. It also provided a public calculation trail that connects architecture development, food inputs, benchmarks, calculations, outputs and sensitivity tests.

A further strength is the separation of quantified essential-nutrient adequacy from broader food-matrix exposure. This allows the model to retain the nutritional significance of diverse whole foods without inventing adequacy benchmarks for compounds that do not yet have them.

### 4.9 Limitations and external-validation programme

The limitations are substantial. The architecture is author-developed and may reflect design judgement that has not been independently replicated. The 44 scenarios were generated and evaluated within the same framework, so they demonstrate internal operability rather than external validation. The food quantities were iteratively constructed to meet prespecified constraints; the high adequacy counts therefore show feasibility of target-pattern construction, not the adequacy of existing population diets. The public supplement does not preserve every intermediate formulation, limiting direct reconstruction of the full development history.

Food-composition values are estimates and can vary by cultivar, season, geography, processing, cooking, edible portion and product formulation. Fortification is brand- and jurisdiction-specific. The model estimated intake rather than absorption or biological status. Iron, zinc, calcium and vitamin D remain especially sensitive to bioavailability, physiology or environmental exposure. The safety screen requires source-form refinement. The model did not evaluate cost, food access, acceptability, cuisine implementation, satiety, adherence, postprandial response, medication interactions, biomarkers, clinical outcomes or long-term behaviour. It cannot be generalized to excluded life stages or clinical populations (28, 29).

External validation should proceed in several stages. First, independent experts should reproduce the failure-mode mapping, domain assignment and boundary decisions. Second, observed dietary records, 24-h recalls, weighed records or national survey data should be mapped into the same food-by-nutrient matrix to test whether the domains predict actual nutrient vulnerabilities. Third, alternative domain partitions and established planning or scoring approaches should be compared for transparency, consistency and adequacy performance. Fourth, product-level fortification variability, food cost, culinary feasibility and real-world adherence should be assessed. Biomarker or clinical research should occur only after the construction and intake-validity stages are established.

### 4.10 Implications and conclusion

The architecture offers a transparent way to ask what must be preserved when dietary preferences change. Its practical sequence is simple even though its calculation layer is detailed: identify the function carried by an excluded food; search first for a compatible food pathway; quantify the result; expose any remaining dependency; screen safety; and test whether the conclusion survives plausible perturbations. This sequence may be useful for research, institutional menu design and software-assisted dietary planning, provided the model is not presented as externally validated before such testing occurs.

In conclusion, the proposed nine-domain architecture produced broad modelled benchmark coverage across 44 final preference-tier scenarios while preserving visibility of conditional vitamin D, iron and zinc watches, post-gap restoration sources, controlled iodine and selenium assumptions, source-form safety issues and broader food-matrix pathways. Mean ordinary coverage was 20.64 of 22 nutrients, with no moderate or major ordinary gaps in the final evaluated scenarios. Because the patterns were deliberately constructed and all scenarios remained sensitive to at least some assumptions, the framework should be interpreted as an auditable planning method and internally stress-tested prototype, not as a validated universal diet, proof of real-world dietary adequacy or evidence of clinical efficacy.

## Data Availability

The original contributions presented in the study are included in the article/Supplementary material, further inquiries can be directed to the corresponding author.
